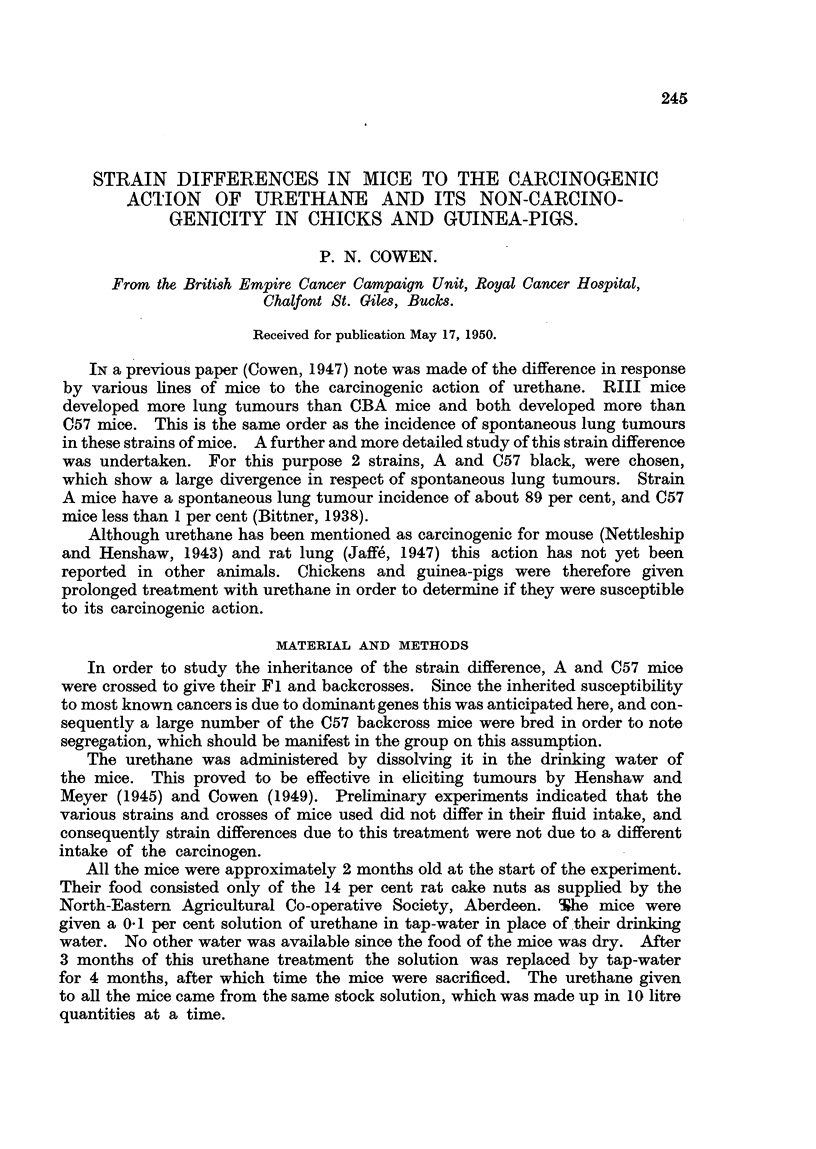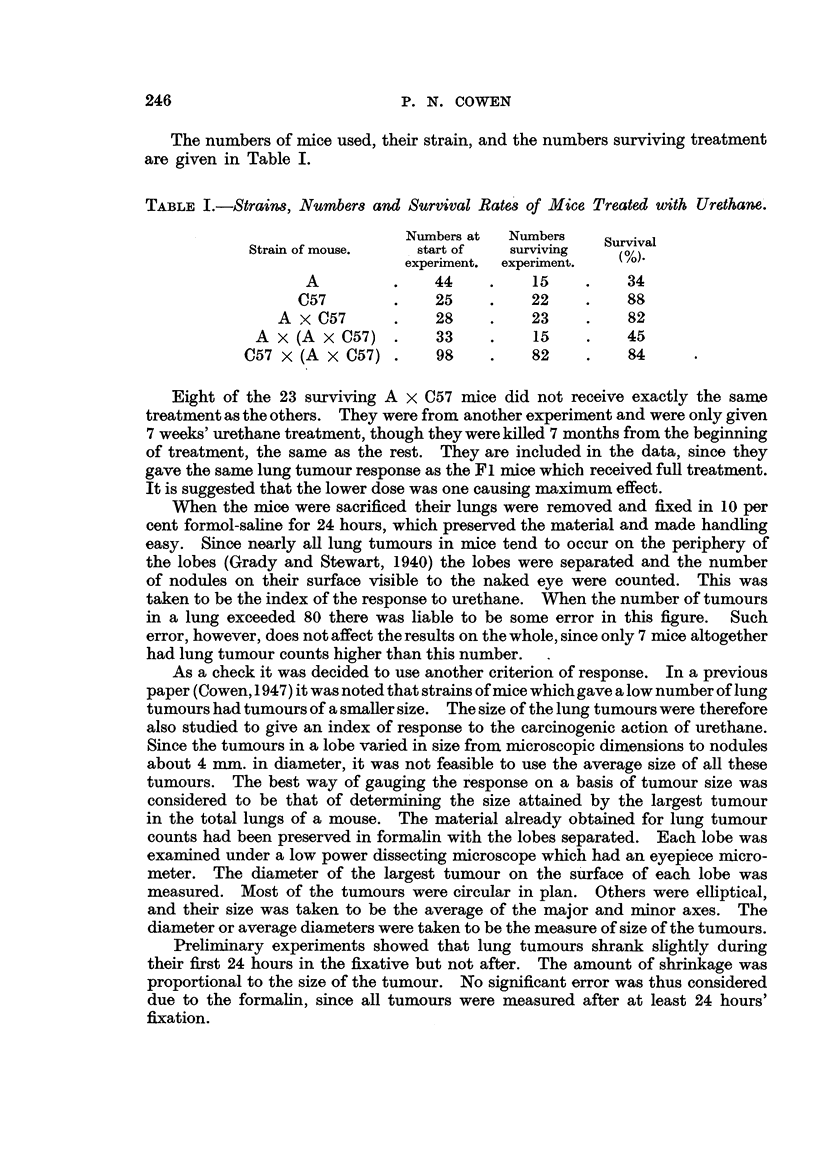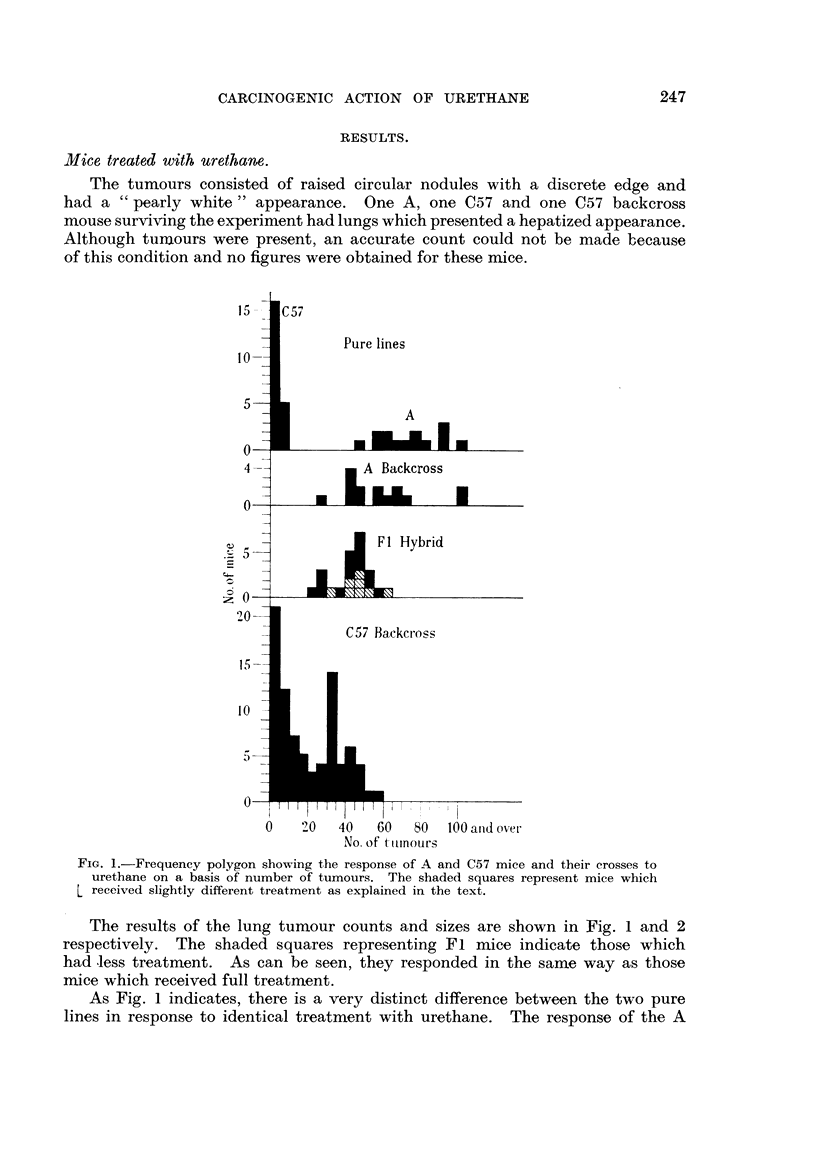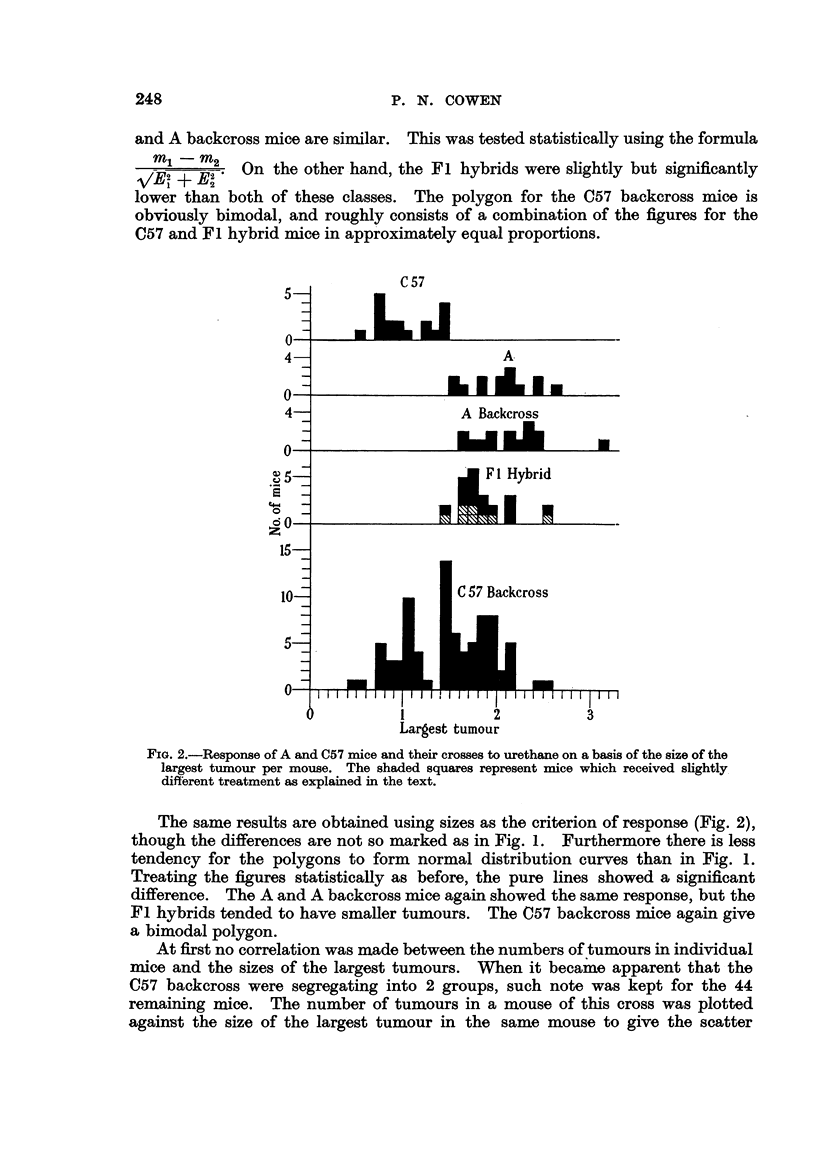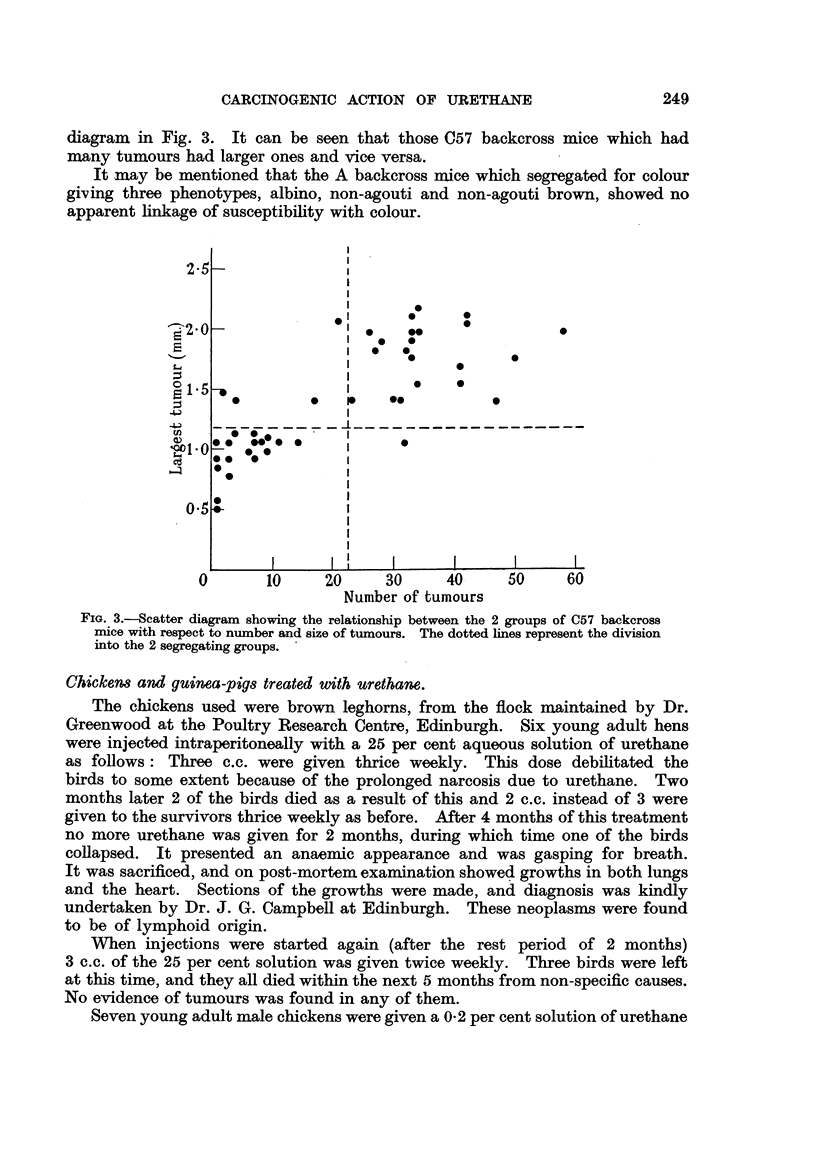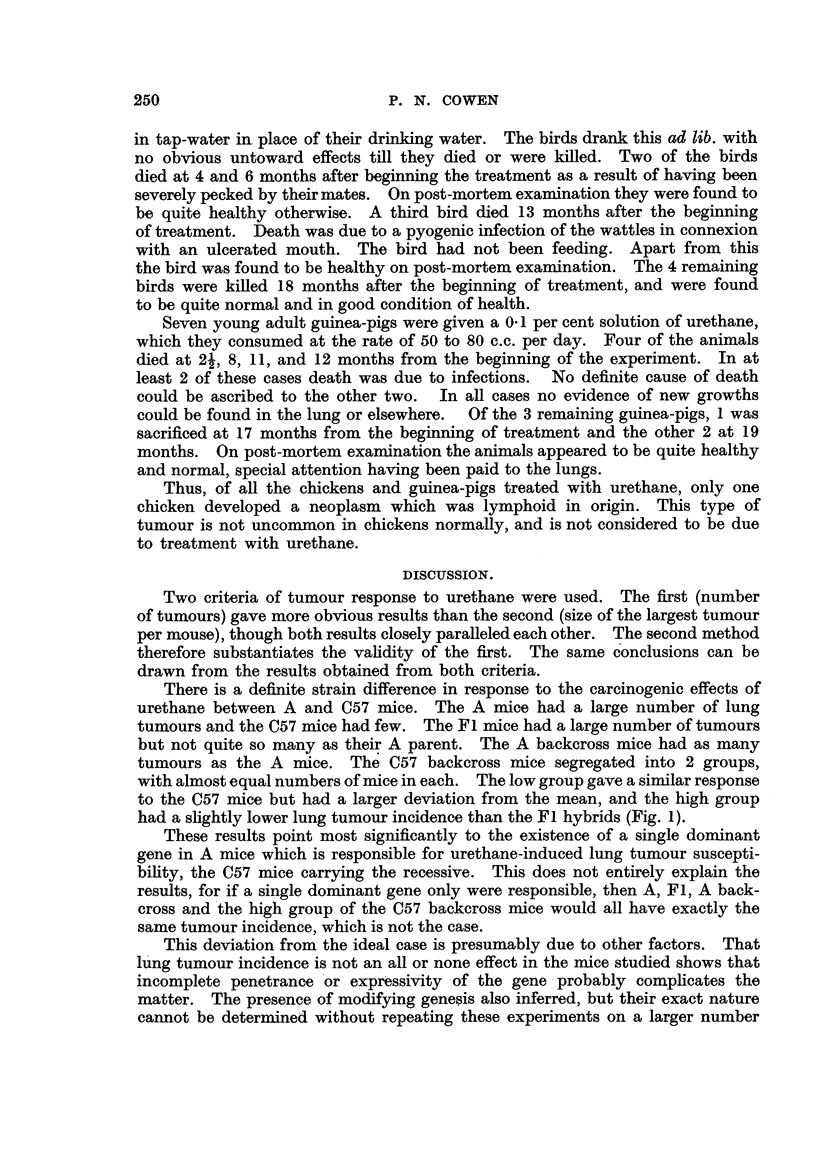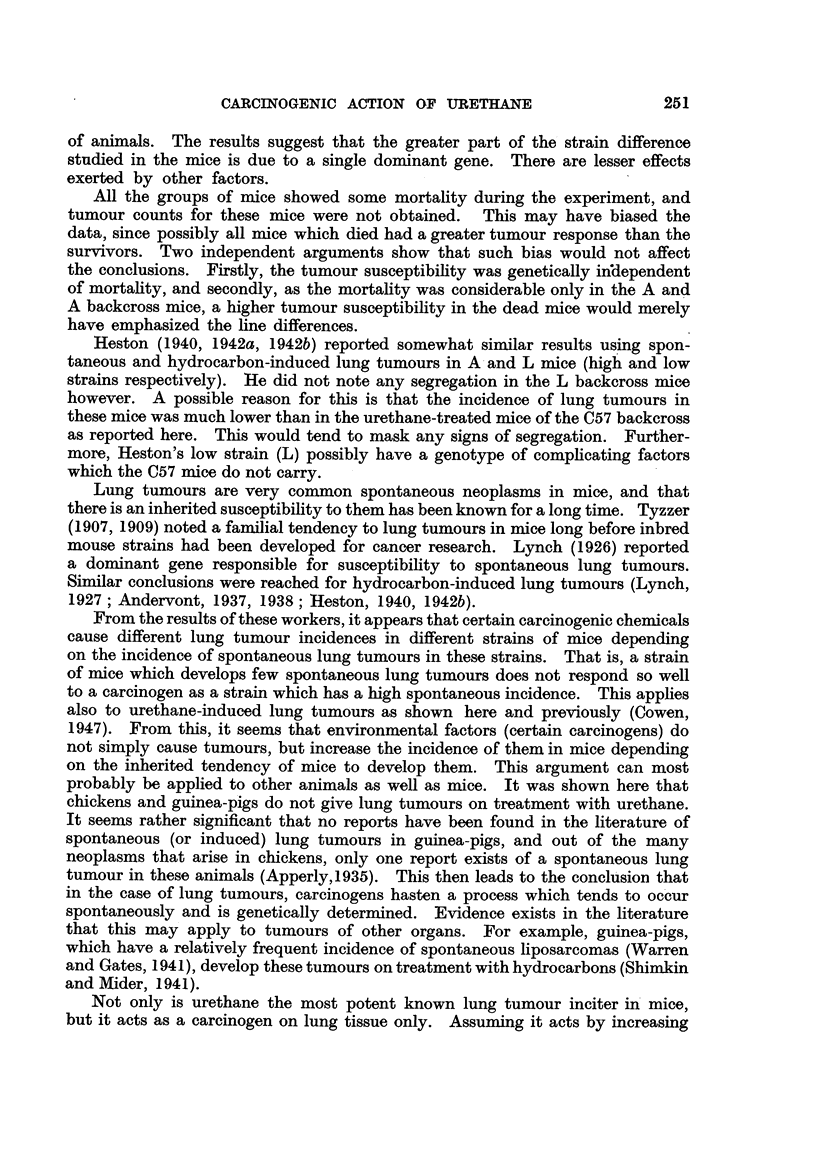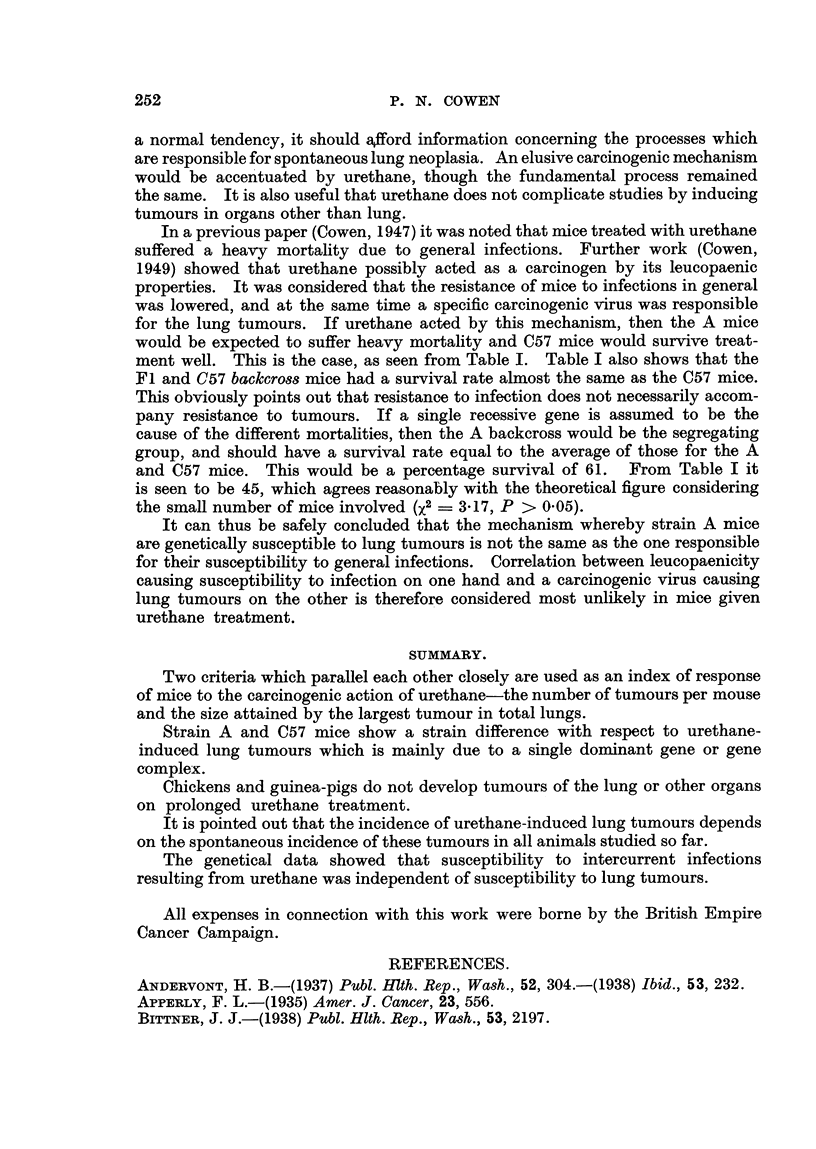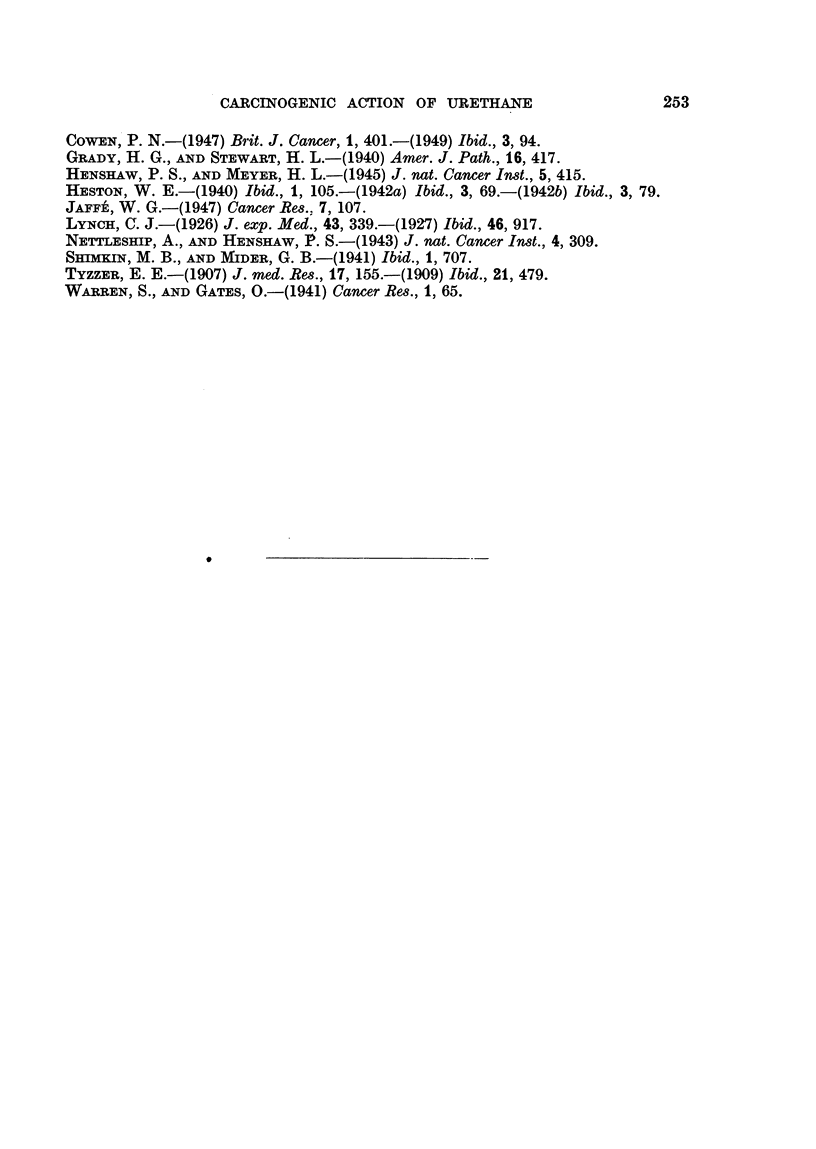# Strain Differences in Mice to the Carcinogenic Action of Urethane and its Non-Carcinogenicity in Chicks and Guinea-pigs

**DOI:** 10.1038/bjc.1950.24

**Published:** 1950-06

**Authors:** P. N. Cowen


### STRAIN DIFFERENCES IN MICE TO THE CARCINOGENIC ACTION OF URETHANE AND ITS NON-CARCINOGENICITY IN CHICKS AND GUINEA-PIGS.

P. N. COWEN.

*From the British Empire Cancer Campaign Unit, Royal Cancer Hospital, Chalfont St. Giles, Bucks.*

Received for publication May 17, 1950.

In a previous paper (Cowen, 1947) note was made of the difference in response by various lines of mice to the carcinogenic action of urethane. RIII mice developed more lung tumours than CBA mice and both developed more than C57 mice. This is the same order as the incidence of spontaneous lung tumours in these strains of mice. A further and more detailed study of this strain difference was undertaken. For this purpose 2 strains, A and C57 black, were chosen, which show a large divergence in respect of spontaneous lung tumours. Strain A mice have a spontaneous lung tumour incidence of about 89 per cent, and C57 mice less than 1 per cent (Bittner, 1938).

Although urethane has been mentioned as carcinogenic for mouse (Nettleship and Henshaw, 1943) and rat lung (Jaffé, 1947) this action has not yet been reported in other animals. Chickens and guinea-pigs were therefore given prolonged treatment with urethane in order to determine if they were susceptible to its carcinogenic action.

### MATERIAL AND METHODS

In order to study the inheritance of the strain difference, A and C57 mice were crossed to give their F1 and backcrosses. Since the inherited susceptibility to most known cancers is due to dominant genes this was anticipated here, and consequently a large number of the C57 backcross mice were bred in order to note segregation, which should be manifest in the group on this assumption.

The urethane was administered by dissolving it in the drinking water of the mice. This proved to be effective in eliciting tumours by Henshaw and Meyer (1945) and Cowen (1949). Preliminary experiments indicated that the various strains and crosses of mice used did not differ in their fluid intake, and consequently strain differences due to this treatment were not due to a different intake of the carcinogen.

All the mice were approximately 2 months old at the start of the experiment. Their food consisted only of the 14 per cent rat cake nuts as supplied by the North-Eastern Agricultural Co-operative Society, Aberdeen. The mice were given a 0·1 per cent solution of urethane in tap-water in place of their drinking water. No other water was available since the food of the mice was dry. After 3 months of this urethane treatment the solution was replaced by tap-water for 4 months, after which time the mice were sacrificed. The urethane given to all the mice came from the same stock solution, which was made up in 10 litre quantities at a time.

The numbers of mice used, their strain, and the numbers surviving treatment are given in Table I.

TABLE I.—*Strains, Numbers and Survival Rates of Mice Treated with Urethane.*

| Strain of mouse. | Numbers at start of experiment. | Numbers surviving experiment. | Survival (%). |
|---|---|---|---|
| A | 44 | 15 | 34 |
| C57 | 25 | 22 | 88 |
| A × C57 | 28 | 23 | 82 |
| A × (A × C57) | 33 | 15 | 45 |
| C57 × (A × C57) | 98 | 82 | 84 |

Eight of the 23 surviving A × C57 mice did not receive exactly the same treatment as the others. They were from another experiment and were only given 7 weeks' urethane treatment, though they were killed 7 months from the beginning of treatment, the same as the rest. They are included in the data, since they gave the same lung tumour response as the F1 mice which received full treatment. It is suggested that the lower dose was one causing maximum effect.

When the mice were sacrificed their lungs were removed and fixed in 10 per cent formol-saline for 24 hours, which preserved the material and made handling easy. Since nearly all lung tumours in mice tend to occur on the periphery of the lobes (Grady and Stewart, 1940) the lobes were separated and the number of nodules on their surface visible to the naked eye were counted. This was taken to be the index of the response to urethane. When the number of tumours in a lung exceeded 80 there was liable to be some error in this figure. Such error, however, does not affect the results on the whole, since only 7 mice altogether had lung tumour counts higher than this number.

As a check it was decided to use another criterion of response. In a previous paper (Cowen, 1947) it was noted that strains of mice which gave a low number of lung tumours had tumours of a smaller size. The size of the lung tumours were therefore also studied to give an index of response to the carcinogenic action of urethane. Since the tumours in a lobe varied in size from microscopic dimensions to nodules about 4 mm. in diameter, it was not feasible to use the average size of all these tumours. The best way of gauging the response on a basis of tumour size was considered to be that of determining the size attained by the largest tumour in the total lungs of a mouse. The material already obtained for lung tumour counts had been preserved in formalin with the lobes separated. Each lobe was examined under a low power dissecting microscope which had an eyepiece micrometer. The diameter of the largest tumour on the surface of each lobe was measured. Most of the tumours were circular in plan. Others were elliptical, and their size was taken to be the average of the major and minor axes. The diameter or average diameters were taken to be the measure of size of the tumours.

Preliminary experiments showed that lung tumours shrank slightly during their first 24 hours in the fixative but not after. The amount of shrinkage was proportional to the size of the tumour. No significant error was thus considered due to the formalin, since all tumours were measured after at least 24 hours' fixation.

RESULTS.

*Mice treated with urethane.*

The tumours consisted of raised circular nodules with a discrete edge and had a "pearly white" appearance. One A, one C57 and one C57 backcross mouse surviving the experiment had lungs which presented a hepatized appearance. Although tumours were present, an accurate count could not be made because of this condition and no figures were obtained for these mice.

FIG. 1.—Frequency polygon showing the response of A and C57 mice and their crosses to urethane on a basis of number of tumours. The shaded squares represent mice which received slightly different treatment as explained in the text.

The results of the lung tumour counts and sizes are shown in Fig. 1 and 2 respectively. The shaded squares representing F1 mice indicate those which had less treatment. As can be seen, they responded in the same way as those mice which received full treatment.

As Fig. 1 indicates, there is a very distinct difference between the two pure lines in response to identical treatment with urethane. The response of the A

and A backcross mice are similar. This was tested statistically using the formula $\frac{m_1 - m_2}{\sqrt{E_1^2 + E_2^2}}$. On the other hand, the F1 hybrids were slightly but significantly lower than both of these classes. The polygon for the C57 backcross mice is obviously bimodal, and roughly consists of a combination of the figures for the C57 and F1 hybrid mice in approximately equal proportions.

FIG. 2.—Response of A and C57 mice and their crosses to urethane on a basis of the size of the largest tumour per mouse. The shaded squares represent mice which received slightly different treatment as explained in the text.

The same results are obtained using sizes as the criterion of response (Fig. 2), though the differences are not so marked as in Fig. 1. Furthermore there is less tendency for the polygons to form normal distribution curves than in Fig. 1. Treating the figures statistically as before, the pure lines showed a significant difference. The A and A backcross mice again showed the same response, but the F1 hybrids tended to have smaller tumours. The C57 backcross mice again give a bimodal polygon.

At first no correlation was made between the numbers of tumours in individual mice and the sizes of the largest tumours. When it became apparent that the C57 backcross were segregating into 2 groups, such note was kept for the 44 remaining mice. The number of tumours in a mouse of this cross was plotted against the size of the largest tumour in the same mouse to give the scatter diagram in Fig. 3. It can be seen that those C57 backcross mice which had many tumours had larger ones and vice versa.

It may be mentioned that the A backcross mice which segregated for colour giving three phenotypes, albino, non-agouti and non-agouti brown, showed no apparent linkage of susceptibility with colour.

FIG. 3.—Scatter diagram showing the relationship between the 2 groups of C57 backcross mice with respect to number and size of tumours. The dotted lines represent the division into the 2 segregating groups.

*Chickens and guinea-pigs treated with urethane.*

The chickens used were brown leghorns, from the flock maintained by Dr. Greenwood at the Poultry Research Centre, Edinburgh. Six young adult hens were injected intraperitoneally with a 25 per cent aqueous solution of urethane as follows: Three c.c. were given thrice weekly. This dose debilitated the birds to some extent because of the prolonged narcosis due to urethane. Two months later 2 of the birds died as a result of this and 2 c.c. instead of 3 were given to the survivors thrice weekly as before. After 4 months of this treatment no more urethane was given for 2 months, during which time one of the birds collapsed. It presented an anaemic appearance and was gasping for breath. It was sacrificed, and on post-mortem examination showed growths in both lungs and the heart. Sections of the growths were made, and diagnosis was kindly undertaken by Dr. J. G. Campbell at Edinburgh. These neoplasms were found to be of lymphoid origin.

When injections were started again (after the rest period of 2 months) 3 c.c. of the 25 per cent solution was given twice weekly. Three birds were left at this time, and they all died within the next 5 months from non-specific causes. No evidence of tumours was found in any of them.

Seven young adult male chickens were given a 0·2 per cent solution of urethane in tap-water in place of their drinking water. The birds drank this *ad lib.* with no obvious untoward effects till they died or were killed. Two of the birds died at 4 and 6 months after beginning the treatment as a result of having been severely pecked by their mates. On post-mortem examination they were found to be quite healthy otherwise. A third bird died 13 months after the beginning of treatment. Death was due to a pyogenic infection of the wattles in connexion with an ulcerated mouth. The bird had not been feeding. Apart from this the bird was found to be healthy on post-mortem examination. The 4 remaining birds were killed 18 months after the beginning of treatment, and were found to be quite normal and in good condition of health.

Seven young adult guinea-pigs were given a 0·1 per cent solution of urethane, which they consumed at the rate of 50 to 80 c.c. per day. Four of the animals died at 2½, 8, 11, and 12 months from the beginning of the experiment. In at least 2 of these cases death was due to infections. No definite cause of death could be ascribed to the other two. In all cases no evidence of new growths could be found in the lung or elsewhere. Of the 3 remaining guinea-pigs, 1 was sacrificed at 17 months from the beginning of treatment and the other 2 at 19 months. On post-mortem examination the animals appeared to be quite healthy and normal, special attention having been paid to the lungs.

Thus, of all the chickens and guinea-pigs treated with urethane, only one chicken developed a neoplasm which was lymphoid in origin. This type of tumour is not uncommon in chickens normally, and is not considered to be due to treatment with urethane.

### DISCUSSION.

Two criteria of tumour response to urethane were used. The first (number of tumours) gave more obvious results than the second (size of the largest tumour per mouse), though both results closely paralleled each other. The second method therefore substantiates the validity of the first. The same conclusions can be drawn from the results obtained from both criteria.

There is a definite strain difference in response to the carcinogenic effects of urethane between A and C57 mice. The A mice had a large number of lung tumours and the C57 mice had few. The F1 mice had a large number of tumours but not quite so many as their A parent. The A backcross mice had as many tumours as the A mice. The C57 backcross mice segregated into 2 groups, with almost equal numbers of mice in each. The low group gave a similar response to the C57 mice but had a larger deviation from the mean, and the high group had a slightly lower lung tumour incidence than the F1 hybrids (Fig. 1).

These results point most significantly to the existence of a single dominant gene in A mice which is responsible for urethane-induced lung tumour susceptibility, the C57 mice carrying the recessive. This does not entirely explain the results, for if a single dominant gene only were responsible, then A, F1, A backcross and the high group of the C57 backcross mice would all have exactly the same tumour incidence, which is not the case.

This deviation from the ideal case is presumably due to other factors. That lung tumour incidence is not an all or none effect in the mice studied shows that incomplete penetrance or expressivity of the gene probably complicates the matter. The presence of modifying genesis also inferred, but their exact nature cannot be determined without repeating these experiments on a larger number of animals. The results suggest that the greater part of the strain difference studied in the mice is due to a single dominant gene. There are lesser effects exerted by other factors.

All the groups of mice showed some mortality during the experiment, and tumour counts for these mice were not obtained. This may have biased the data, since possibly all mice which died had a greater tumour response than the survivors. Two independent arguments show that such bias would not affect the conclusions. Firstly, the tumour susceptibility was genetically independent of mortality, and secondly, as the mortality was considerable only in the A and A backcross mice, a higher tumour susceptibility in the dead mice would merely have emphasized the line differences.

Heston (1940, 1942*a*, 1942*b*) reported somewhat similar results using spontaneous and hydrocarbon-induced lung tumours in A and L mice (high and low strains respectively). He did not note any segregation in the L backcross mice however. A possible reason for this is that the incidence of lung tumours in these mice was much lower than in the urethane-treated mice of the C57 backcross as reported here. This would tend to mask any signs of segregation. Furthermore, Heston's low strain (L) possibly have a genotype of complicating factors which the C57 mice do not carry.

Lung tumours are very common spontaneous neoplasms in mice, and that there is an inherited susceptibility to them has been known for a long time. Tyzzer (1907, 1909) noted a familial tendency to lung tumours in mice long before inbred mouse strains had been developed for cancer research. Lynch (1926) reported a dominant gene responsible for susceptibility to spontaneous lung tumours. Similar conclusions were reached for hydrocarbon-induced lung tumours (Lynch, 1927 ; Andervont, 1937, 1938 ; Heston, 1940, 1942*b*).

From the results of these workers, it appears that certain carcinogenic chemicals cause different lung tumour incidences in different strains of mice depending on the incidence of spontaneous lung tumours in these strains. That is, a strain of mice which develops few spontaneous lung tumours does not respond so well to a carcinogen as a strain which has a high spontaneous incidence. This applies also to urethane-induced lung tumours as shown here and previously (Cowen, 1947). From this, it seems that environmental factors (certain carcinogens) do not simply cause tumours, but increase the incidence of them in mice depending on the inherited tendency of mice to develop them. This argument can most probably be applied to other animals as well as mice. It was shown here that chickens and guinea-pigs do not give lung tumours on treatment with urethane. It seems rather significant that no reports have been found in the literature of spontaneous (or induced) lung tumours in guinea-pigs, and out of the many neoplasms that arise in chickens, only one report exists of a spontaneous lung tumour in these animals (Apperly,1935). This then leads to the conclusion that in the case of lung tumours, carcinogens hasten a process which tends to occur spontaneously and is genetically determined. Evidence exists in the literature that this may apply to tumours of other organs. For example, guinea-pigs, which have a relatively frequent incidence of spontaneous liposarcomas (Warren and Gates, 1941), develop these tumours on treatment with hydrocarbons (Shimkin and Mider, 1941).

Not only is urethane the most potent known lung tumour inciter in mice, but it acts as a carcinogen on lung tissue only. Assuming it acts by increasing a normal tendency, it should afford information concerning the processes which are responsible for spontaneous lung neoplasia. An elusive carcinogenic mechanism would be accentuated by urethane, though the fundamental process remained the same. It is also useful that urethane does not complicate studies by inducing tumours in organs other than lung.

In a previous paper (Cowen, 1947) it was noted that mice treated with urethane suffered a heavy mortality due to general infections. Further work (Cowen, 1949) showed that urethane possibly acted as a carcinogen by its leucopaenic properties. It was considered that the resistance of mice to infections in general was lowered, and at the same time a specific carcinogenic virus was responsible for the lung tumours. If urethane acted by this mechanism, then the A mice would be expected to suffer heavy mortality and C57 mice would survive treatment well. This is the case, as seen from Table I. Table I also shows that the F1 and *C57 backcross* mice had a survival rate almost the same as the C57 mice. This obviously points out that resistance to infection does not necessarily accompany resistance to tumours. If a single recessive gene is assumed to be the cause of the different mortalities, then the A backcross would be the segregating group, and should have a survival rate equal to the average of those for the A and C57 mice. This would be a percentage survival of 61. From Table I it is seen to be 45, which agrees reasonably with the theoretical figure considering the small number of mice involved ($\chi^2 = 3{\cdot}17$, $P > 0{\cdot}05$).

It can thus be safely concluded that the mechanism whereby strain A mice are genetically susceptible to lung tumours is not the same as the one responsible for their susceptibility to general infections. Correlation between leucopaenicity causing susceptibility to infection on one hand and a carcinogenic virus causing lung tumours on the other is therefore considered most unlikely in mice given urethane treatment.

### SUMMARY.

Two criteria which parallel each other closely are used as an index of response of mice to the carcinogenic action of urethane—the number of tumours per mouse and the size attained by the largest tumour in total lungs.

Strain A and C57 mice show a strain difference with respect to urethane-induced lung tumours which is mainly due to a single dominant gene or gene complex.

Chickens and guinea-pigs do not develop tumours of the lung or other organs on prolonged urethane treatment.

It is pointed out that the incidence of urethane-induced lung tumours depends on the spontaneous incidence of these tumours in all animals studied so far.

The genetical data showed that susceptibility to intercurrent infections resulting from urethane was independent of susceptibility to lung tumours.

All expenses in connection with this work were borne by the British Empire Cancer Campaign.

### REFERENCES.

ANDERVONT, H. B.—(1937) *Publ. Hlth. Rep., Wash.*, **52**, 304.—(1938) *Ibid.*, **53**, 232.
APPERLY, F. L.—(1935) *Amer. J. Cancer*, **23**, 556.
BITTNER, J. J.—(1938) *Publ. Hlth. Rep., Wash.*, **53**, 2197.

Cowen, P. N.—(1947) *Brit. J. Cancer*, **1**, 401.—(1949) *Ibid.*, **3**, 94.
Grady, H. G., and Stewart, H. L.—(1940) *Amer. J. Path.*, **16**, 417.
Henshaw, P. S., and Meyer, H. L.—(1945) *J. nat. Cancer Inst.*, **5**, 415.
Heston, W. E.—(1940) *Ibid.*, **1**, 105.—(1942*a*) *Ibid.*, **3**, 69.—(1942*b*) *Ibid.*, **3**, 79.
Jaffé, W. G.—(1947) *Cancer Res.*, **7**, 107.
Lynch, C. J.—(1926) *J. exp. Med.*, **43**, 339.—(1927) *Ibid.*, **46**, 917.
Nettleship, A., and Henshaw, P. S.—(1943) *J. nat. Cancer Inst.*, **4**, 309.
Shimkin, M. B., and Mider, G. B.—(1941) *Ibid.*, **1**, 707.
Tyzzer, E. E.—(1907) *J. med. Res.*, **17**, 155.—(1909) *Ibid.*, **21**, 479.
Warren, S., and Gates, O.—(1941) *Cancer Res.*, **1**, 65.